# Error correction due to background subtraction in ratiometric calcium measurements with CCD camera

**DOI:** 10.1016/j.heliyon.2020.e04180

**Published:** 2020-06-24

**Authors:** Vyacheslav M. Shkryl

**Affiliations:** Department of Biophysics of Ion Channels, Bogomoletz Institute of Physiology, 4 Bogomoletz Street, Kyiv, 01024, Ukraine

**Keywords:** Neuroscience, Mathematical modeling, Biophysics, Cellular neuroscience, Physiology, Fluorescence, Ca^2+^, Ratiometric method, Fura-2, Grynkiewitz's formula, CCD camera, Neuron

## Abstract

**Background:**

Ca^2+^ plays an important role in many physiological processes and an accurate study of these signals is important. In modern fluorescence microscopy, a charge-coupled device (CCD) camera is widely deployed for calcium imaging. The ratiometric method is used for the fluorescence dye Fura-2 and Grynkiewitz's formula (Grynkiewicz et al., 1985) is commonly used to convert fluorescence to free Ca^2+^ concentration ([Ca^2+^]). But the need to subtract the background signal can lead to a big error in ratiometric calcium measurements. When the error due to background subtraction occurs, the fluorescence ratio of 340 nm divided by 380 nm lights may be twice as large as the actual value. Under conditions when the excitation intensity is not adjusted to ensure the same throughput of the objective lens for ultraviolet dye illumination, the indicator does not gradually bleach out for channels with a wavelength of 340 nm and 380 nm light, which lead to an additional error in determining the concentration of Ca^2+^.

**New method:**

Here we present a new approach for calculating [Ca^2+^] from the ratiometric fluorescence of Fura-2 dye imaged by a CCD camera. It is designed to optimize [Ca^2+^] measurements with photobleaching correction without background subtraction error. A mathematical method is also provided for removing the existing underestimated value of fluorescence at an excitation wavelength of 340 nm and compensating for the bleaching rate for both channels with wavelengths of 340 nm and 380 nm using a power function.

**Results:**

In cultured neurons, the calculations of the free Ca^2+^ concentration during Ca^2+^ transients estimated by the old and new methods, determine it to the same extent. This comparison was made under conditions without errors through background subtraction. If there is this error, the old method calculates [Ca^2+^] with a much higher, rather than the actual value.

**Conclusions:**

We present a modified Grynkiewitz's formula for calculation [Ca^2+^] for ratiometric dye, such as Fura-2 imaged by a CCD camera, with photobleaching correction without background subtraction error.

## 1 Introduction

The calcium ion (Ca^2+^) is a common second messenger that regulates different physiological pathways as secretion, fertilization, gene transcription, and apoptosis (Berridge et al., 2000; Pozzan et al., 1994). Tsien et al. were introduced Ca^2+^ fluorescent indicators for studies of Ca^2+^ signaling (Tsien et al., 1982). Studies of cellular Ca^2+^ signaling have been greatly facilitated by the availability of fluorescent Ca^2+^ indicators, which suitable to monitor Ca^2+^ signals inside a cell. In its simple form, the measurement of [Ca^2+^] using a fluorescent indicator requires only an appropriate light source and a photomultiplier tube (PMT) detector. A PMT was used for many years and still used when high sensitivity, fast measuring are needed. The contemporary imaging system uses a low-light intensified charge-coupled device (CCD) camera, with an array of isolated elements (or pixels), attached to the fluorescence microscope. Currently, two approaches are widely used to assess the dynamics of free intracellular Ca^2+^: single-wavelength and two-wavelength (ratiometric) methods. The ratiometric method is used not only for quantitative measurements of the concentration of free Ca^2+^ ([Ca^2+^]) but also for qualitative analysis, such as single-wavelength techniques, valuable for relative changes in Ca^2+^ (Grynkiewicz et al., 1985; Minta et al., 1989). Processing the ratio data of two-wavelength emission considerably reduces the effects of uneven dye loading, dye leakage, and photobleaching.

Fura-2 is a ratiometric fluorescence indicator of Ca^2+^. The largest dynamic range was detected at an excitation wavelength of 340 nm and 380 nm that make the wavelengths preferred for [Ca^2+^] measurements. The Grynkiewitz's formula is commonly used to convert PMT ratiometric fluorescence into [Ca^2+^] for the dye Fura-2 (Grynkiewicz et al., 1985).

Continuous excitatory illumination often leads to irreversible destruction or photobleaching of the fluorophore. Loss of fluorescence during data acquisition (photobleaching or bleaching) results in an error in determining the concentration of free Ca^2+^ (Scheenen et al., 1996). Using the ratiometric method, the effect of photobleaching is solved by dividing the fluorescence at 340 nm by the correspondent signal at 380 nm light. The background should be also subtracted from each excitation wavelength before determining the final ratio. Using the Grynkiewitz's formula, subtracting the background value from an individual element of the *x-y* fluorescence images can lead to a large error in determining the concentration of free Ca^2+^.

In this work, we verify the Grynkiewicz's formula of calculation of [Ca^2+^] for CCD cameras when background subtraction error appears. And a new approach was proposed for calculating the concentration of the free calcium ions with photobleaching correction, using fluorescent excitation channels at 340 and 380 nm lights, which can be used for the ratiometric dye as the Fura-2.

## 2 Materials and methods

All experimental procedures were performed in accordance with international principles of the European Convention for the protection of vertebrate animals used for experimental and other scientific purposes (European convention, Strasburg, 1986); the Law of Ukraine “On protection of animals from cruelty” and approved by the Animal Care Committee of Bogomoletz Institute of Physiology.

### 2.1 Cell culture and solutions

All experiments were performed on cultured neurons of the hippocampus of newborn Wistar rats. The primary culture of hippocampal neurons was prepared as previously reported (Shkryl et al., 1999). Ca^2+^ imaging studies were carried out in 7–14 days of cultivation. A cover glass with cells was placed in the solution included in mM: NaCl – 140.0; KCl – 2.0; СaCl_2_ – 2.0; MgCl_2_ - 2.0; HEPES – 10.0; pH = 7.4. To induce Ca^2+^ transient was used as a depolarization solution. Depolarization solution containing 50.0 mM KCl was equivalent to bath solution described above except that part of NaCl was replaced by KCl. All chemicals were obtained from Sigma-Aldrich (St. Louis, MO).

Before the experiment, the cells were loaded with 5 μM Fura-2 acetoxymethyl ester (Fura-2 AM) for 30 min at 37 °C and an additional 20 min for deesterification of the dye. For [Ca^2+^] calculation we used vitro value of Fura-2 Kd = 224 nM (Grynkiewicz et al., 1985). Ca^2+^ transients were induced by a 5-seconds application of the depolarization solution with high potassium solution thereby opening voltage-sensitive Ca^2+^ channels in the cell membrane. To apply solutions we used a perfusion system, controlled by a computer and synchronized by data acquisition software.

### 2.2 Calcium imaging

In our study, we used a CCD camera (Olympus XM10) mounted on an Olympus IX71 inverted microscope equipped with an Olympus LUCPlanFFN 20x/0.45 lens and MT10 illumination system that includes a filter wheel exchanger (340 and 380 nm) and 150W xenon arc burner. Cell M software (Olympus, Japan) was used for data collection. The acquisition speed for one data point (340 nm and 380 nm) was 1.3 Hz.

### 2.3 Data analysis

Data analysis was performed using the IDL programming environment (ITT Visual Information Solutions). In the recorded images with excitation of 340 nm and 380 nm lights, the region of interest (ROI) was selected based on the criteria for the signal difference at the peak of Ca^2+^ transient and at its basal level. This allows us to make a good selection of the soma area. For the subsequent analyses, ROI was selected on the soma of a neuron without a nuclear region. All experiments were performed at room temperature (22–25 °C). Data are presented as mean ± SEM.

## 3 Results

The emission fluorescence from the Fura-2 indicator does not have a linear relationship to the [Ca^2+^] and the dye fluorescence provides a relative indication of the magnitude of the Ca^2+^ signal, but can be calibrated. The Grynkiewicz formula with taking into account the background level is:

(1) $$\left[{\text{Ca}}^{\;2+}\right]=K_{d}\frac{\left(\frac{{\text{F}}_{340}-\text{Bgr}}{{\text{F}}_{380}-\text{Bgr}}-{\text{R}}_{\text{min}}\right)}{\left({\text{R}}_{\text{max}}-\frac{{\text{F}}_{340}-\text{Bgr}}{{\text{F}}_{380}-\text{Bgr}}\right)}\frac{{\text{F}}_{380}^{max}-\text{Bgr}}{{\text{F}}_{380}^{min}-\text{Bgr}}$$

where ${\text{R}}_{\text{min}}=\frac{{\text{F}}_{340}^{min}-\text{Bgr}}{{\text{F}}_{380}^{max}-\text{Bgr}};$${\text{R}}_{\text{max}}=\frac{{\text{F}}_{340}^{max}-\text{Bgr}}{{\text{F}}_{380}^{min}-\text{Bgr}}$; *K*_*d*_ – dissociation constant; Bgr – background level; F_340_, F_380_ – fluorescence at 340 nm and 380 nm excitation wavelength; ${\text{F}}_{340}^{min}$ and ${\text{F}}_{340}^{max}$or ${\text{F}}_{380}^{min}$ and ${\text{F}}_{380}^{max}$- minimal and maximal values of F_340_ and F_380_ respectively (Grynkiewicz et al., 1985). From this equation is clear that the real value of calcium concentration could be correctly determined then ${\text{F}}_{380}-\text{Bgr}>0$.

Using CCD camera signals are recorded of two-dimensional information as an image (*x, y*) or dynamic recording in time array (*x-y-t*). The size of the PMD detector and one CCD pixel is three orders of magnitude, making the probability of detecting a signal at the level of one CCD pixel much lower than that of the PMT. On the other hand, the quantum efficiency is better for scientific CCDs to compare to PMT, especially those with semiconductor photo-cathodes, can detect single photons. CCDs are not capable of doing that. Unlike the signal recorded using a PMT, the CCD camera, at an individual pixel, records the reduced amount of photons with a high noise level at each data point of the *x-y* image (Heintzmann, 2006; Jerome, 2011). The necessary subtraction of the background signal, performed from each pixel of the *x-y* array images at excitation of 340 and 380 nm lights, can have the resulting values as a negative or a value of zero, which may cause an error in calculating of [Ca^2+^].

**Figure 1:**
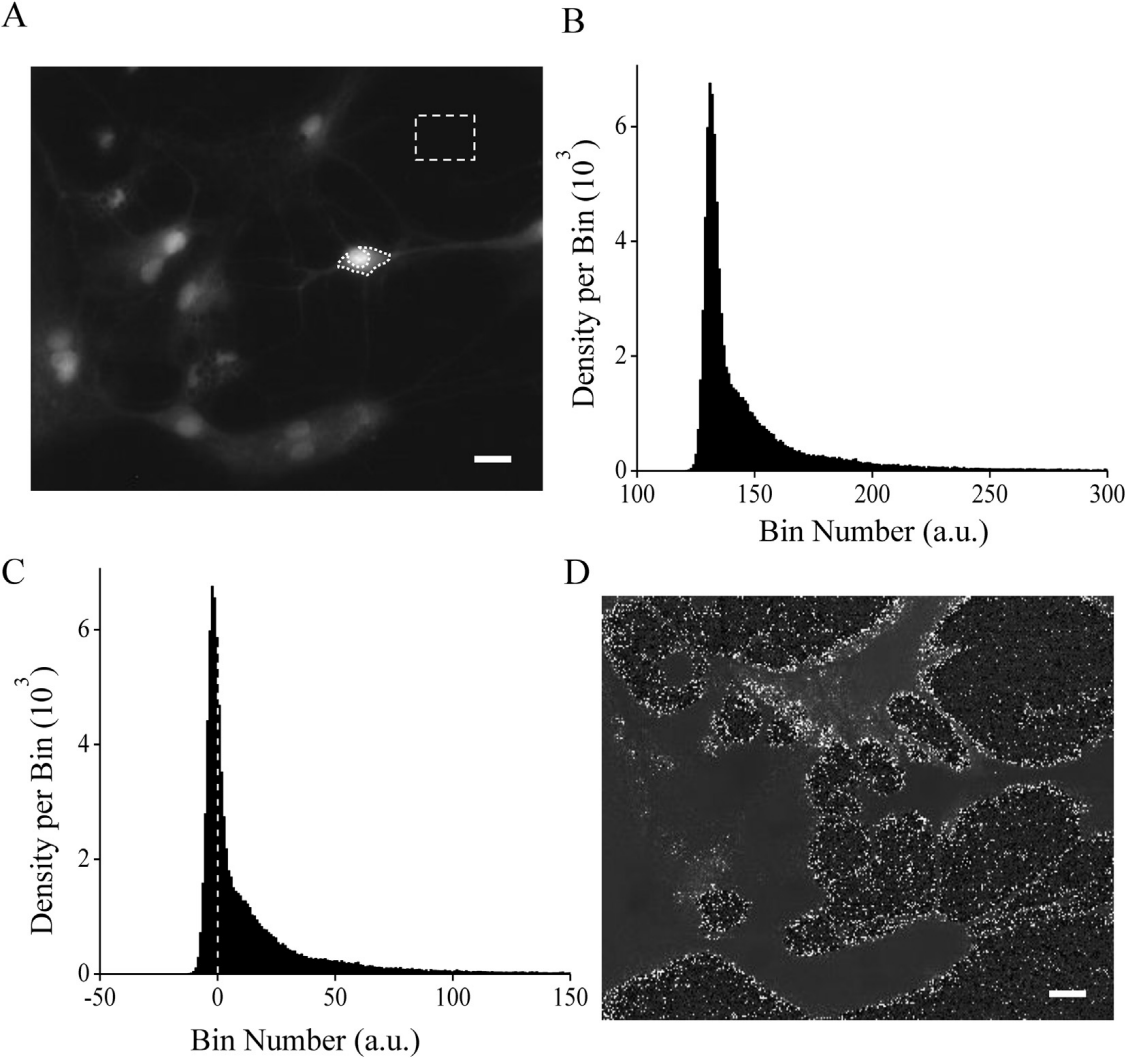
Intracellular Ca^2+^ measurement using the Fura-2 dye in cultured neurons of the hippocampus. A: Grayscale image displays the Fura-2 emission signal at 380 nm excitation wavelength (F_380_) in rest. The neuron ROI (dotted white line) was selected on the soma of the neuron without a nuclear region. The dashed rectangle represents the place outside the neuron where the background level was obtained. The histogram of F_380_ pixel intensity distribution before and after subtracting the background is shown in Fig. 1B and 1C, respectably. The dashed line represents the zero value. D: Grayscale image of the distorted ratio of F_340_/F_380_ fluorescence obtained after the background subtraction. Scale bar is 20 μm.

A background signal can be caused by endogenous compounds that are unevenly distributed in the cell, such as collagen fibers, pyridine nucleotides, which absorb and emit light at wavelengths very similar to Ca^2+^ indicators (Bootman et al., 2013a). Using 50–100 μM Fura-2 autofluorescence of the cell may be as large as 5% - 10 % of total fluorescence after dye loading (Neher, 2013). During ratiometric measurements, the background subtraction is an important step for the correctly estimating of [Ca^2+^] (Bootman et al., 2013a). Figure 1 shows an experiment in which Fura-2-loaded cells recorded in resting conditions. Figure 1A demonstrates a selected grayscale *x-y* image of a fluorescent signal at 380 nm light.

The histogram of the intensity distribution of F_380_ at one frame is shown in Figure 1B. The minimum value of F_380_ was 117 a. u., and for F_340_ was 120 a. u. The mean background level of 133 a. u. in the given image area (dashed line) was subtracted from each pixel of the *x-y* image. After subtracting the background level, the histogram of the signal intensity distribution was obtained, which is shown in Figure 1C. The peak of the histogram was -2.9 intensity values. As can be seen, after subtracting the background level, the fluorescent signal had both negative and zero values. After subtracting the background level, the image of the ratio of 340 nm–380 nm signals ($\frac{{\text{F}}_{340}}{{\text{F}}_{380}}$) is shown in Figure 1D. As you can see, the signal is highly distorted and many values have infinity values (white pixels) since division by zero occurred. After background subtraction, the ratio ($\frac{{\text{F}}_{340}}{{\text{F}}_{380}}$) was distorted. The background value was also measured inside the soma of neurons before Fura-2 loading cells with the same conditions and fluorescence setting. It was 130.7 ± 0.6 at 340 nm and 133.7 ± 0.9 at 380 nm (n = 6) excitation wavelengths.

**Figure 2:**
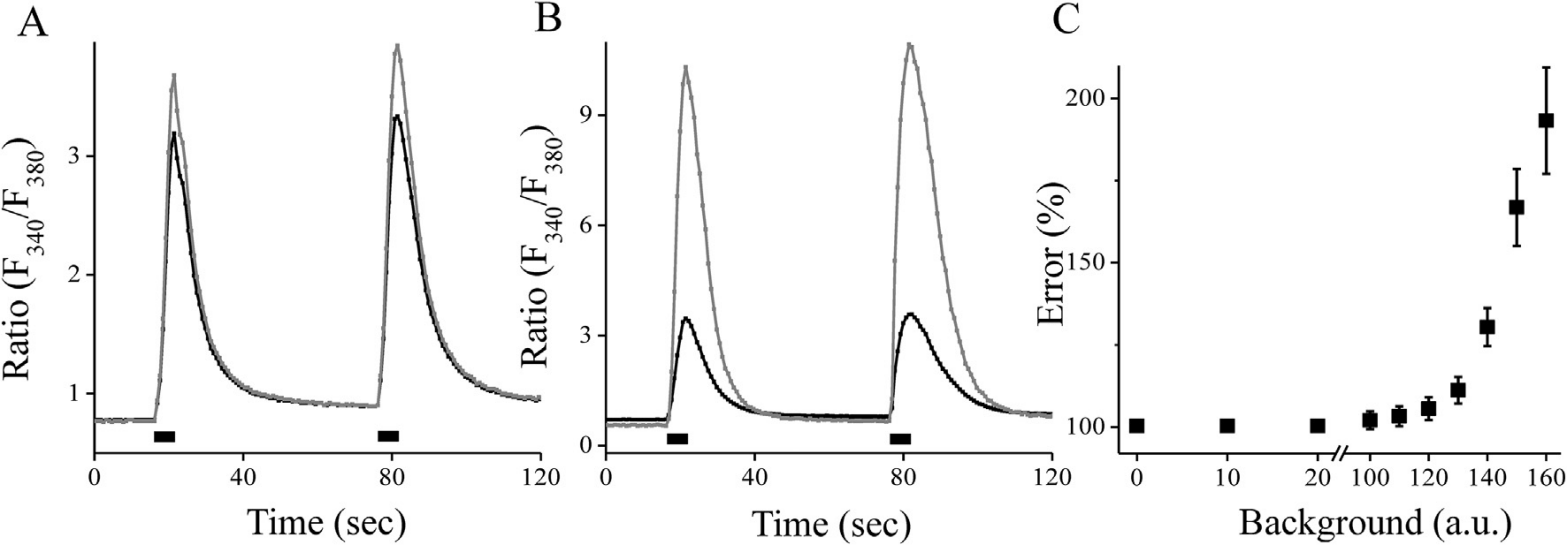
The appearance of an error in the ratio of F_340_/F_380_ signals after subtracting the background level. The figure shows an experiment in which Fura-2-loaded hippocampal neurons. A, B: representative traces of the Fura-2 ratio ($\frac{{\text{F}}_{340}}{{\text{F}}_{380}}$) of Ca^2+^ transients under the condition of subtracting the background of 140 units (111114_c13#1) and 180 units (111114_c7#1), respectively. The gray line corresponds to the condition when the background value was subtracted from each pixel of fluorescence of *x-y* image; the black line – the background value was subtracted from averaged value from selected ROI of the neuron. The black rectangular indicates the application of a depolarization solution for 5 s. C: The error in determining the value of the ratio ($\frac{{\text{F}}_{340}}{{\text{F}}_{380}}$) on the value of the background. The error was calculated in 8 cells as a percentage value of Ca^2+^ transient amplitude (*dR*) obtained with each pixel subtraction divided on *dR* with averaged subtraction of the background level.

We consider two scenarios for subtracting the background level: the value is subtracted from each data point (pixel) of an *x-y* array of images for 340 nm and 380 nm fluorescence (each-pixel subtraction) or the average signal at selected ROI on the images (the averaged subtraction). The averaged subtraction is analogous to the measurement of a PMT detector. To show this consequence we check the value of the ratio signal recorded in cultured neurons loaded with Fura-2, AM dye. Two Ca^2+^ transients were recorded at soma area of neuron caused by 5-seconds application of the depolarization solution. The ratio $\frac{{\text{F}}_{340}-\text{Bgr}}{{\text{F}}_{380}-\text{Bgr}}$ is obtained then the background of 140 units was subtracted at each-pixel (a gray line) and form the averaged signal from the ROI (a black line), shown in Figure 2A. For each recording, the ratios were calculated from the same cell area. The value of 140 units was obtained at a site outside of the cell. Figure 2B shows another recording and the ratio with subtracting the background level of 180 units. As can be seen, the signals differ even at a lower background level and considerably by 180 units. As can be seen from the presented experimental data, the subtraction of the background level, from each pixel, leads to an error in determining the value of the ratio of fluorescence of 340 nm to 380 nm excitation wavelengths. According to the histogram of the distribution of the pixel intensity (Figure 1B), the signal at light of 380 nm was not underestimated. To describe the error in ratio that appears then the background is subtracted, the amplitude of Ca^2+^ transient for various background values were calculated. The amplitude of the first Ca^2+^ transient (*dR*) was calculated as the amplitude of the ratio at the peak of the transient from the base level when using each-pixel and the average background subtraction methods. How can we see from Figure 2C that the significant error occurs when the background level is above 120 a. u. This value is lower for the background level outside the cell that was 133.7 ± 0.4 units (n = 8). To reduce the difference in signals level, all the data presented in this experiment were obtained on the same day using the same settings of a fluorescent system, as well as and the exposure time and intensity of excitation. Of the 8 tested cells, without background subtraction, the basal levels of fluorescent signals at soma of neuron were 261.8 ± 12.7 at 380 nm and 226.4 ± 8.0 at 340 nm. The peak value of Ca^2+^ transient was 178.5 ± 5.7 at 380 nm and 279.3 ± 12.9 at 340 nm correspondently. In the case of each-pixel background subtraction signal at 380 nm excitation has a greater tendency towards a negative or zero value and can lead to errors in calculating the real Ca^2+^ signal not only at resting level but also at peak Ca^2+^ transient, that produce an error to determine the amplitude of it. To get the result without the error, it is necessary to average over the cell area or ROI of it and only then subtract the background level.

As mentioned earlier, [Ca^2+^] can be determined by a single wavelength method, but there will be no online dye bleaching correction. The calibration equation for a single wavelength can be written as:

(2) $$\left[{\text{Ca}}^{\;2+}\right]=K_{d}\frac{\left(\text{F}-{\text{F}}_{\text{min}}\right)}{\left({\text{F}}_{\text{max}}-\text{F}\right)}$$

where F_min_ and F_max_ minimal and maximal fluorescence values (Grynkiewicz et al., 1985).

For ratiometric imaging, additional adjustment is required. It is that the excitation intensity at each wavelength is adjusted to match the resultant fluorescence to the bit-depth of the system (Bootman et al., 2013a). Using a chamber with indicator dye or some fluorescence grass, the fluorescence intensities for both wavelengths can be adjusted so that they are the same, attenuating the power level for excitation light at 380 nm. In our experiment, it was found that such conditions correspond to a 2.5-fold decrease in the light intensity at 380 nm compared with excitation at 340 nm.

**Figure 3:**
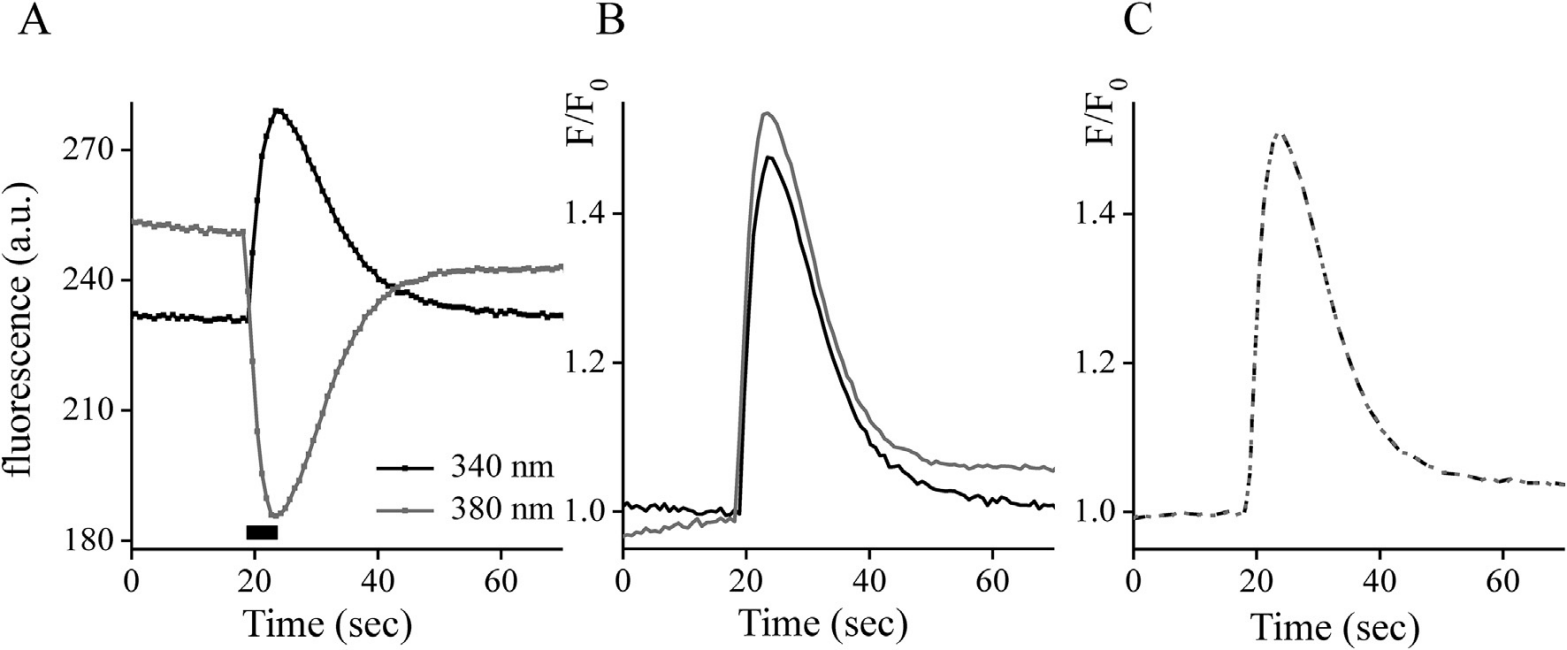
Fluorescence of Fura-2-loaded cells recorded during Ca^2+^ transient. Panel A depicts the measurement of fluorescence at excitation wavelengths of 340 nm (black line) and 380 nm (gray line). The black rectangular indicates the application of a depolarization solution. B: normalized fluorescent signals at both excitation wavelength, see the main text for additional explanations. A black line corresponds normalized fluorescence at 340 nm excitation wavelengths; a gray line – at 380 nm. C: normalized bleaching-free signal at excitation wavelengths of 340 nm (black dash) and 380 nm (gray short dash). The coefficient of the lost excitation power energy, *C*_*0*_ was equal to 1.01. Ca^2+^ transient was caused by the 5-seconds application of the depolarization solution.

A wavelength at < 360 nm, fluorescence increases when the Ca^2+^ ion binds to the dye, and decreases at > 360 nm. A fluorophore such as Fura-2 when excited at 340 nm and 380 nm wavelengths should have similar kinetic properties. Are the relative change values of these signals the same or different? It is well known that upon Ca^2+^ binding to the Fura-2 dye molecule the fluorescence emission differs mirror-like at excitation wavelengths of 340 and 380 nm, which are represented by Ca^2+^ transient (Figure 3A).

To compare relative change in fluorescence the signal at 380 nm is represented as the normalized signal $2-\frac{{\text{F}}_{380}}{{\text{F}}_{380}\left(0\right)}$ and compared with the normalized signal at 340 nm as ${\left(\frac{{\text{F}}_{340}}{{\text{F}}_{340}\left(0\right)}\right)}^{C_{0}}$, where F_380_(0) and F_340_(0) are the initial, baseline fluorescence; where *C*_*0*_ is the coefficient of the lost excitation power energy during the passage of light through the objective lens at a wavelength of 340 nm compared to 380 nm. Under the condition when fluorescent outputs coincide *C*_*0*_ is 1. Interestingly, these signals are almost identical, except for the distorted part due to the dye bleaching effect (Figure 3B).

A dye bleaching function (γ) can be found as the averaged difference between these normalized signals added to 1:

(3) $$\text{γ}=\;\frac{1}{2}\left({\left(\frac{{\text{F}}_{340}}{{\text{F}}_{340}\left(0\right)}\right)}^{C_{0}}+\frac{{\text{F}}_{380}}{{\text{F}}_{380}\left(0\right)}\right)$$

The coefficient *С*_*0*_ can be determined under specified boundary. Then for the given values of F_340_ and F_380_, we can write: ${\left(\frac{{\text{F}}_{340}(1)}{{\text{F}}_{340}\left(0\right)}\right)}^{C_{0}}+\frac{{\text{F}}_{380}(1)}{{\text{F}}_{380}\left(0\right)}=2$, where F_340_(1) and F_380_(1) is the value of Ca^2+^ transient at peak (or defined [Ca^2+^]). Then we can find the coefficient as:

(4) $$C_{0}=\frac{\ln\left(2-\frac{{\text{F}}_{380}(1)}{{\text{F}}_{380}\left(0\right)}\right)}{\ln\frac{{\text{F}}_{340}(1)}{{\text{F}}_{340}\left(0\right)}}$$

**Figure 4:**
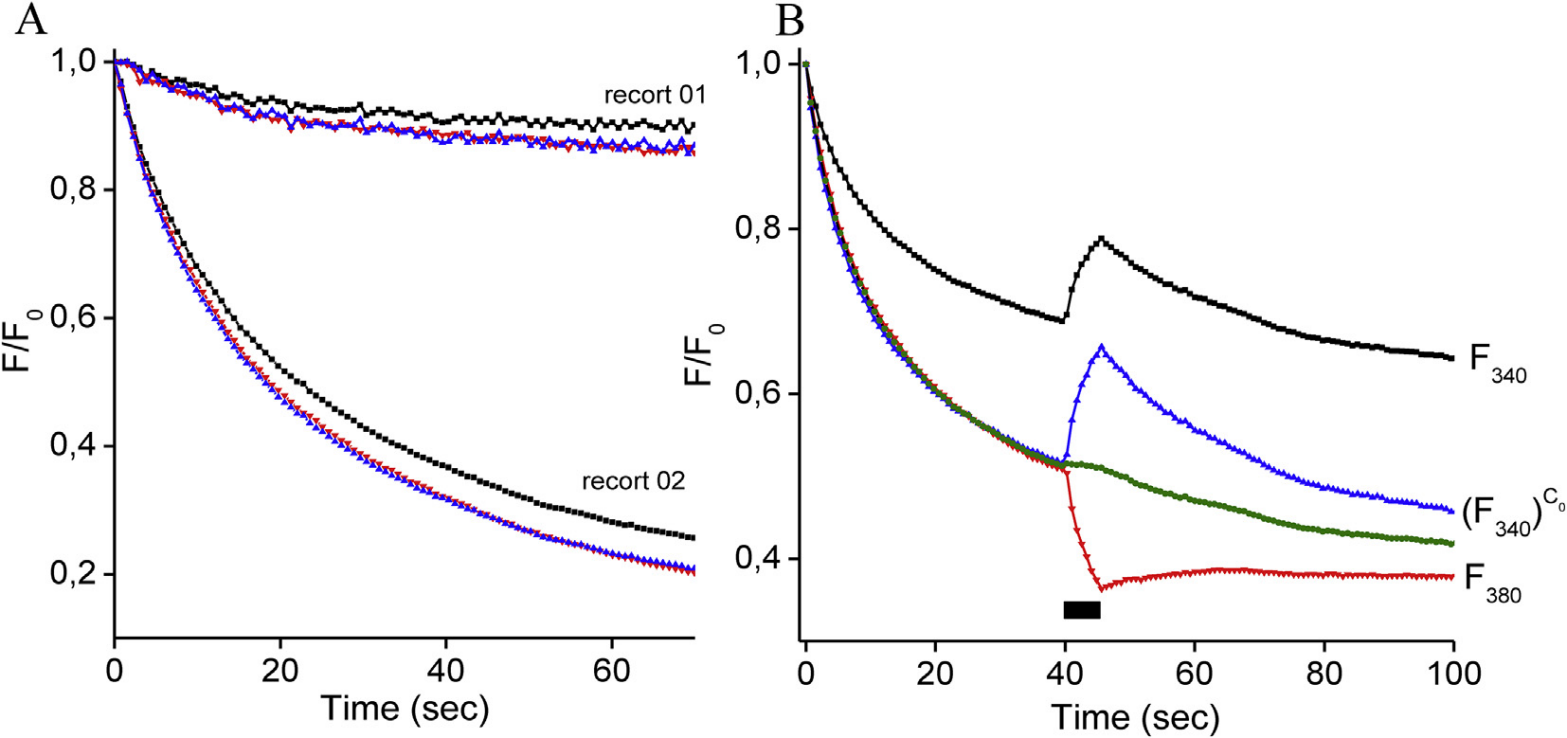
Bleached fluorescence of Fura-2-loaded cells. Panel A presents two cell recordings of the fluorescence emission at excitation wavelengths of 340 nm (black square line) and 380 nm (red triangle line) upon continuous illumination. During record 02, power energy of emission light at 340 nm and 380 nm was increased compared to the record 01. For both records, the blue triangle line presents the fluorescence emission at F_340_ recalculated as ${\left(\frac{{\text{F}}_{340}}{{\text{F}}_{340}\left(0\right)}\right)}^{C_{0}}$ with *C*_*0*_ = 1.35 for the record 01 and *C*_*0*_ = 1.15 for the record 02. The fluorescence was normalized to 1 at the starting point of experiments (time = 0 s). Panel B depicts the fluorescence at excitation wavelengths of 340 nm (black square line) and 380 nm (red triangle line) during Ca^2+^ transient. The blue triangle line presents the fluorescence emission at F_340_ recalculated as ${\left(\frac{{\text{F}}_{340}}{{\text{F}}_{340}\left(0\right)}\right)}^{C_{0}}$ with *C*_*0*_ = 1.77. The green line is the bleaching function obtained by Eq. (3). Black rectangular indicates the application of a depolarization solution. Ca^2+^ transient was caused by the 5-seconds application of the depolarization solution.

In case when the excitation intensity at each wavelength does not match the resultant fluorescence to the bit-depth of the fluorescence system the bleaching functions for a wavelength of 340 nm and 380 nm lights can be different. Figure 4 shows the kinetics of fluorescence emission at the two wavelengths with continuous illumination. At the beginning of the experiment, the fluorescence intensity for both at wavelengths was normalized to 100%. Figure 4A shows record without any intervention, just how the fluorescence drop-in time. The 380 traces dropped more rapidly than the 340 traces in both cases with a low and high bleaching rate. The best way to correct the fluorescence difference is power 340 trace with the power *C*_*0*_ as ${\left(\frac{{\text{F}}_{340}}{{\text{F}}_{340}\left(0\right)}\right)}^{C_{0}}$. The resulting signals much bleaching rate with 380 traces in both records. Figure 4B presents another record during the recording of Ca^2+^ transient. In this example bleaching rate of F_340_ (black square line) and F_380_ (red triangle line) was differed significantly, but after correction as ${\left(\frac{{\text{F}}_{340}}{{\text{F}}_{340}\left(0\right)}\right)}^{C_{0}}$ bleaching rate much both wavelengths excitation (red and blue triangle lines). As can be seen from this figure the green trace (the dye bleaching function, obtained by formula 3) reflects the decrease in the traces of fluorescence of light at 380 nm and corrected 340 nm. The rate of bleaching effect can be simple mathematically corrected for 340 traces using power function with the coefficient of the lost excitation power energy *C*_*0*_ which can be calculated by Eq. (4) with a calibration.

The bleaching-free normalized signals for two wavelengths can be presented as: $\text{F}1\;\left(340\right)=\frac{1}{\text{γ}}{\left(\frac{{\text{F}}_{340}}{{\text{F}}_{340}\left(0\right)}\right)}^{C_{0}}\text{ and F}2\;\left(380\right)=\left(2-\frac{{\text{F}}_{380}}{{\text{F}}_{380}\left(0\right)\cdot \text{γ}}\right)$. As can be seen from Figure 3C, the normalized signals F1 and F2 are almost identical. The bleaching function γ obtained by Eq. (3) removes the component of the bleaching dye. The bleaching-free signals can be found as: $\frac{{\left({\text{F}}_{340}\right)}^{C_{0}}}{\text{γ}}$ and $\frac{{\text{F}}_{380}}{\text{γ}}$ at 340 nm and 380 nm of excitation wavelength respectively.

Averaging ${\left(\frac{{\text{F}}_{340}}{{\text{F}}_{340}\left(0\right)}\right)}^{C_{0}}\;$ and $\left(2-\frac{{\text{F}}_{380}}{{\text{F}}_{380}\left(0\right)}\right)$signals compensates the dye bleaching effect. Also, the resulting bleaching free fluorescence can be found by the next equation:

(5) $$\text{F}=\;\frac{1}{2}\left({\left(\frac{{\text{F}}_{340}}{{\text{F}}_{340}\left(0\right)}\right)}^{C_{0}}-\frac{{\text{F}}_{380}}{{\text{F}}_{380}\left(0\right)}\right)+1$$

Multiplying Eq. (5) by ${\text{F}}_{340}\left(0\right)$ or ${\text{F}}_{380}\left(0\right)$ allows comparing the fluorescent signals from other cells.

(6) $$\text{F}={\text{F}}_{340}\left(0\right)\cdot \left(\frac{1}{2}\left({\left(\frac{{\text{F}}_{340}}{{\text{F}}_{340}\left(0\right)}\right)}^{C_{0}}-\frac{{\text{F}}_{380}}{{\text{F}}_{380}\left(0\right)}\right)+1\right),\text{or }={\text{F}}_{380}\left(0\right)\text{·}\left(\frac{1}{2}\left(\frac{{\text{F}}_{340}}{{\text{F}}_{340}\left(0\right)}-{\left(\frac{{\text{F}}_{380}}{{\text{F}}_{380}\left(0\right)}\right)}^{1/C_{0}}\right)+1\right)$$

The coefficient of the lost excitation power energy, *C*_*0*_ could be found using Eq. (4). There is an option to use Fura-2 just at 380 nm (>360 nm) as a single wavelength method.

Rewrite formula 2 in condition as$\text{F}=2-\frac{{\text{F}}_{380}}{{\text{F}}_{380}\left(0\right)}$, ${\text{F}}_{max}=2-\frac{{\text{F}}_{380}^{min}}{{\text{F}}_{380}\left(0\right)}$, and ${\text{F}}_{min}=2-\frac{{\text{F}}_{380}^{max}}{{\text{F}}_{380}\left(0\right)}$ then:

(7) $$\left[{\text{Ca}}^{\;2+}\right]=\frac{\left({\text{F}}_{380}^{max}-{\text{F}}_{380}\right)}{\left({\text{F}}_{380}-{\text{F}}_{380}^{min}\right)}K_{d}$$

The formula 7 can be used to calculate the concentration of free Ca^2+^ for excitation wavelength >360 nm.

Eq. (6) calculates a new fluorescent signal from F_340_ and F_380_ with compensation for the bleaching dye effect. It can be used for a two-wave excitation method with Fura-2 dye or its analogs. The concentration of free Ca^2+^ can be obtained using Eq. (2) for fluorescence <360 nm or Eq. (7) for >360 nm excitation wavelength. The error through background subtraction that was found for the ratiometric signal is missing for the fluorescence F obtained by Eq. (6). Delivered Eq. (6) collapses the ratio measurement onto a single wavelength without background subtraction error. To calculate [Ca^2+^] using Eq. (2) or 7, the background subtraction step can be skipped, as this value is equally excluded in the numerator and denominator of Eq. (2) or 7.

**Figure 5:**
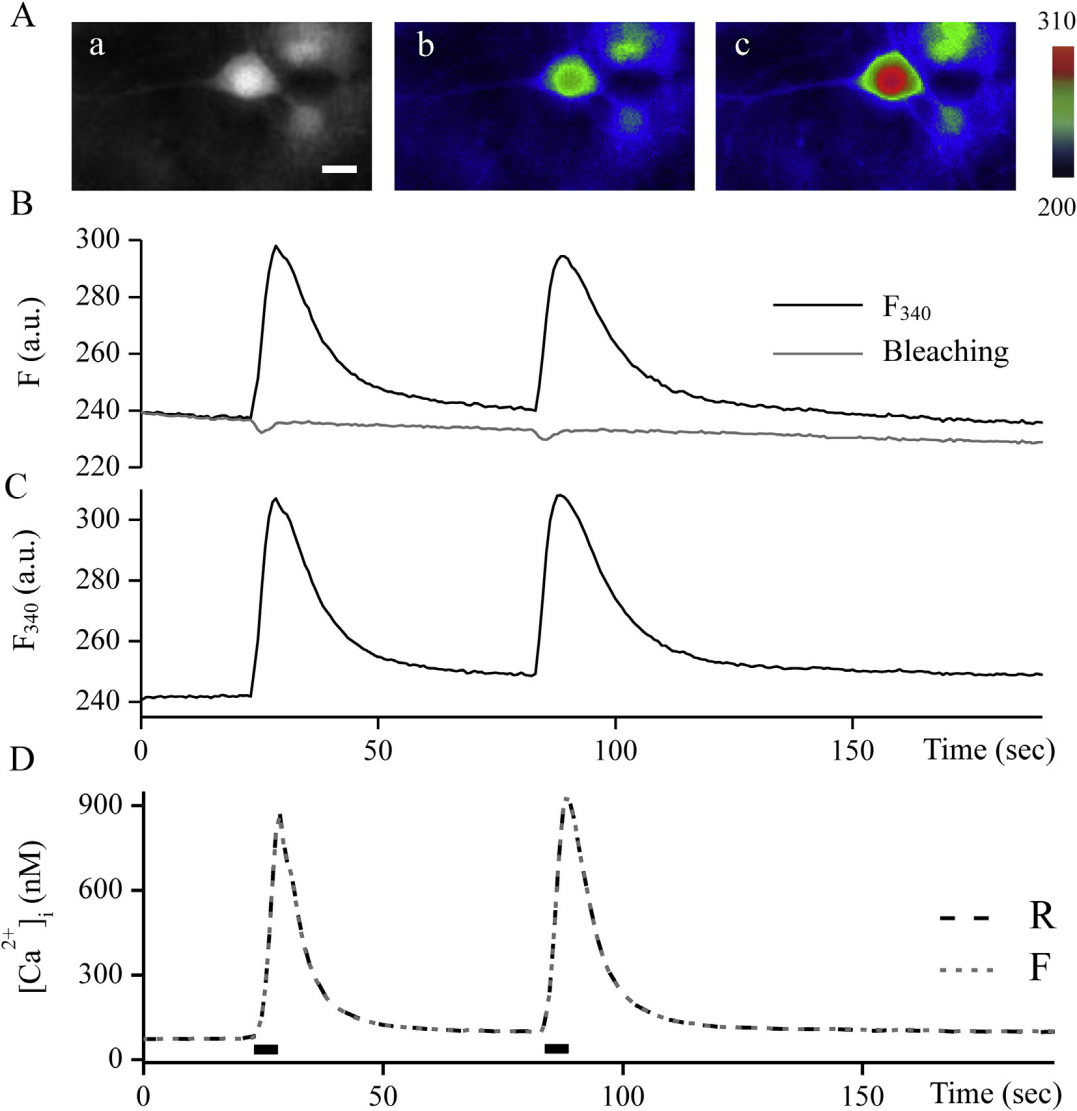
F_340_ and bleaching signals of Fura-2-loaded a hippocampal neuron. Aa: a part of the original fluorescent image at 340 nm light, at rest. It shows a pyramidal neuron. Ab and Ac are pseudocolor fluorescent images obtained by formula 6 at resting condition and at time of the peak of the first Ca^2+^ transient respectively. The color scale represents arbitrary fluorescence intensity values. B: The black line of the trace represents an original fluorescence signal at 340 nm excitation wavelength recorded from the soma of the neuron during two Ca^2+^ transients. The gray line is the bleaching function obtained by Eq. (3). C: The fluorescence at 340 nm light after bleaching correction obtained by Eq. (6). D: The concentration of free Ca^2+^ during two Ca^2+^ transients calculated using formulas 1 for the ratio of F_340_/F_380_ (R; black line) and fluorescence F obtained by formula 6 and 2 (gray line). Ca^2+^ transients were caused by the 5-seconds application of the depolarization solution. The black rectangular indicates the application of the depolarization solution. Scale bar is 20 μm.

Figure 5A a represents an *x-y* grayscale image showing the Fura-2 emission signal at 340 nm light at rest before any manipulation. As can be seen in Figure 5B, the gray trace (the dye bleaching function, obtained by formula 3) reflects the decrease in the black trace of fluorescence of light at 340 nm and well defines the Fura-2 bleaching effect. In Figure 5C shows fluorescence at an excitation wavelength of 340 nm, obtained by Eq. (6). It is free bleaching fluorescence signal. Figure 5Ab and 5Ac show pseudo-color images of the two-dimensional signal obtained by Eq. (6) at rest and the peak of the first Ca^2+^ transient. These are not destroyed signals.

Under conditions without an error due to background subtraction, the calculation of the concentration of free Ca^2+^ using the Grynkiewicz formula 1 and the new technique presented in this paper, formula 6 and 2, should have the same result. For these comparisons, a background value was subtracted from F_340_ and F_380_ by the averaged subtraction circumstance. For both methods, the boundary values of the signals were obtained, as shown in AppendixA. ${\text{F}}_{340}^{min}$ was 74.9 and ${\text{F}}_{340}^{max}$= 195.5; ${\text{F}}_{380}^{min}$ = 20.7 and ${\text{F}}_{380}^{max}$ = 169.4. The values of R_min_ and R_max_ were calculated, which amounted to 0.44 and 9.44, respectively. From the same data record, substituting these values into formula 2 for the resulting fluorescence F and Grynkiewicz formula 1 for the ratio signal *R* concentration of free Ca^2+^ was plotted in Figure 5D. As can be seen, both calculations equally determine [Ca^2+^].

A dye bleaching function can be also written as.

(8) $$\text{γ}=\;\frac{{{\text{F}}_{340}}^{C}+{\text{F}}_{380}}{{{\text{F}}_{340}}^{C}\left(0\right)+{\text{F}}_{380}\left(0\right)}$$

where *C* is the coefficient of the lost excitation power energy for fluorescence F_340_ compare to F_380_. This coefficient can be determined under given boundary conditions of F_340_(1) and F_380_(1) at given [Ca^2+^] or peak of Ca^2+^ transient. From Eqs. (3) and (8), we can write the equation to obtain a value of *C*:

(9) $$2\cdot {\text{F}}_{340}^{\text{C}}\left(1\right)+2\cdot {\text{F}}_{380}\left(1\right)+\;\left({\left(\frac{{\text{F}}_{340}\left(1\right)}{{\text{F}}_{340}\left(0\right)}\right)}^{C_{0}}+\frac{{\text{F}}_{380}\left(1\right)}{{\text{F}}_{380}\left(0\right)}\right)\text{·}\left({\text{F}}_{340}^{\text{C}}\left(0\right)-{\text{F}}_{380}\left(0\right)\right)=0$$

where *C*_*0*_ can be determined by Eq. (4) under the same conditions. The coefficient *C* can be empirically estimated by substituting a value from 0.5 to 2 units in Eq. (9).

The coefficient *C* can be used to calculate ratio value with compensation of different power energy lost during the passage of light through the objective lens at a wavelength of 340 nm compared to 380 nm.

(10) $$R=\frac{{{\text{F}}_{340}}^{C}}{{\text{F}}_{380}}$$

The ratio obtained by Eq. (10) does not affect the lost excitation power for a wavelength of 340 nm light. The ratio should be used to compare the fluorescence ratio of Ca^2+^ signals from different recordings.

## 4 Calculation of [Ca^2+^] without error due to background subtraction

• The excitation intensity at each wavelength need to be adjusted to match the resultant fluorescence to the bit-depth of the system (Bootman et al., 2013a);
• Imaging data is acquired as a time series and must be saved into two image sequences separated into data at 340 nm and 380 nm excitation wavelengths (F_340_ and F_380_);
• Finding a background value at noncellular region;
• Make time profile of fluorescence for two channels F_340_ (*t*) and F_380_ (*t*) at selected ROI of a cell where need to calculate [Ca^2+^];
• Subtract the background from F_340_ and F_380_ signals. The background value must be subtracted from the averaged value of the fluorescence at selected ROI of the cell;
• Obtain ratio of F_340_ and F_380_;
• Using boundary values of fluorescence with the background subtraction (obtained how described in (Barreto-Chang and Dolmetsch, 2009; Bootman et al., 2013b)) calculate [Ca^2+^] by Grynkiewicz formula 1.

To obtain two-dimensional data of [Ca^2+^] (*x-y*), the following protocol can be used:

• Get fluorescence *x-y* image at an excitation wavelength of 340 nm or 380 nm with compensating the effect of bleaching of the dye under Eq. (6);
• Do not need to subtract the background value;
• Using fluorescence boundary values, calculate [Ca^2+^] by Eq. (2) or 7 for wavelengths of 340 nm or 380 nm respectively;

If excitation intensity at the excitation wavelength of 340 nm and 380 nm lights does not correspond to the resulting fluorescence to the bit-depth of the system, mathematical manipulation using a power function removes the difference in bleaching function and fluorescence value at excitation wavelength 340 nm. The coefficient *C*_*0*_ and *C* can be calculated during calibration under boundary conditions using Eqs. (4) and (9).

The obtained *x-y* image [Ca^2+^] can be used for flux analyses, the protocol of which is described in detail in (Neher, 1995; Rios et al., 1999; Shkryl, 2017).

## 5 Discussion

The Fura-2 indicator, developed by Roger Tsien and co-authors (Grynkiewicz et al., 1985), was cited in thousands of scientific papers and is commonly used to study the role of calcium in cell regulation. As mentioned above, the ability to make ratio measurements with Fura-2 allows us to accurately measure the intracellular Ca^2+^ concentration. The ratiometric method removes the photobleaching of the dye and reduces the effect of the uneven loading of the dye. Without the ratiometric method, this is almost impossible online, however, some bleaching function can be obtained by an additional record that can be applied to the data or simply mathematically corrected because it follows a predictable exponential decay (Thomas et al. 2000). In this paper, we obtain Eq. (3), which finds the photobleaching function γ from F_340_ and F_380_ signals. This function can be used to obtain bleaching free fluorescence. In our experiments, the best bleaching curve approximation was achieved by power function x^c^. A x^c^ curve fitting was performed from the baseline value using $\text{γ}=1-a\cdot x^{b}$, where *b* depends on the amount of excitation power, in our experimental conditions takes a value around 0.8. (data is not shown). As can be seen from Figure 5 before Ca^2+^ transient begins to increase there is a slight decrease in the level with a subsequent increase of the bleaching function γ. These changes are due to the slow temporal resolution, which was 0.76 s, while when fluorescence changes during Ca^2+^ transient signal F_380_ are ahead of the F_340_. To reduce this artifact and noise before applying the bleaching function it can be approximated by $1-a\cdot x^{b}$ generated to the same experimental condition.

As mentioned above, background subtraction is an important step for the ratiometric method. Unlike the signal recorded using a PMT, the CCD camera, at an individual pixel, records the reduced amount of photons with a high noise level at each data point of the *x-y* image (Heintzmann, 2006; Jerome, 2011). Also subtracting the background level directly from each pixel of the *x-y* image may be cases where the value is negative or even zero, which naturally leads to distortion of the ratio of signals at 340 nm to 380 nm excitation wavelengths and can lead to a significant error in determining the concentrations of free Ca^2+^ using the Grynkiewicz formula (formula 1 (Grynkiewicz et al., 1985);) that was shown here. When an error occurs due to background subtraction, the ratio may be twice as large as the actual value (Figure 2D). Barreto-Chang O.L. and Dolmetsch R.E. described the protocol of calcium imaging where the procedure of background value subtraction applied from each pixel in the field. They apply an additional step to reduce noise need to adjust the threshold values for each wavelength to generate a ratio image that includes only the cells and not the background and that is not noisy in the region close to the edges of the cells (Barreto-Chang and Dolmetsch, 2009). In the protocol described in this work using a CCD camera to reduce noise and eliminate the error requires averaging the signal from ROI before subtracting the background. To obtain a two-dimensional image of [Ca^2+^] need to use the fluorescence obtained by formula 6.

The resulting fluorescence F obtained by formula 6 is the average value of the normalized signals for the excitation lights of the dye at 340 nm and 380 nm. Due to the different bandwidths of the objective lens for these wavelengths, the normalized fluorescence may differ slightly for 340 nm and 380 nm. Also, the difference between the signals can be because the excitation and emission peak of NADH is 340 nm and 440–470 nm, respectively (Harbig et al., 1976; Paddle, 1985) and its fluorescence contaminates the true UV-exited Fura-2 indicator fluorescence.

Becker and Fay had shown that a bleached form of Fura-2 is still fluorescent and it is not sensitive to calcium over the same range as Fura-2 and ratio method are not completely remove proportional decreasing the concentration of dye (Becker and Fay, 1987). They suggested that to minimize photodegradation, one should decrease the illumination dose and O_2_ concentration. In this way, some errors can appear in calcium concentration measurements. Significant photodegradation of the fluorescence dye leads to an underestimation of the analyze concentrations depending on the intensity and duration of illumination. Scheenen et all shown that the problem can be avoided by including cell-permeant antioxidants such as Trolox in the bathing solution (Scheenen et al., 1996).

During ratiometric Ca^2+^ imaging, in conditions when excitation intensity at each wavelength is not adjusted to match the resultant fluorescence to the bit-depth of the system, UV illumination of a dye not gradually bleaches the indicator for both excitation channels (Becker and Fay, 1987; Bootman et al., 2013a). In this work, it was shown that the existing difference in the dye bleaching function for F_340_ and F_380_ can be mathematically eliminated (by power function) both with a low and high power intensity of the dye excitation and with a significant difference in the dye bleaching rate. The coefficient of the lost excitation power energy, *C*_*0*_ could be simply calculated by Eq. (4) during calibration with known [Ca^2+^] or at a value of baseline and peak value of Ca^2+^ transient. It will help to analyses data if an adjustment of excitation intensity both ratio channels was not much the resultant fluorescence to the bit-depth of the fluorescence system. It removes one trace drooped more rapidly for resulting in not distorted the fluorescence ratio.

Another problem that arises due to the poor performance in fluorescent imaging through ultraviolet excitation is the distortion of the ratio of the F_340_ to F_380_ signals, which restricts comparing this ratio for the different fluorescent settings. Using the coefficient *C,* calculated according to Eq. (9), we can calculate the fluorescence ratio using formula 10 without the limitation.

The advantage of Fura-2 is that it has a good cross-section for two-photon calcium imaging (Wokosin et al., 2004). The Eq. (2) could be used to calculate [Ca^2+^] for two-photon at 680 nm (or <360 nm at a single wavelength excitation). The new Eq. (7) can be used for the two-photon excitation method at 760 nm (or with single wavelength at 380 nm light) and allow you to use Fura-2 (>360 nm) as a dye with excitation at a single wavelength and can serve as an alternative to fluorescent systems with weak UV transmission lens.

## 6 Conclusions

In this paper, we present a modified formula for calculation of free Ca^2+^ concentration for ratiometric dye, such as Fura-2, which recalculates the fluorescence of two channels, imaged by a CCD camera, at excitation wavelengths of 340 nm and 380 nm into fluorescence with photobleaching correction without an error due to background subtraction. The resulting fluorescence can be converted into a concentration of free Ca^2+^. Also, a mathematical elimination technique using the power function is provided to remove existing differences in the dye bleaching rate and fluorescence intensity. It helps compensate the signal at a wavelength of 340 nm light if the excitation intensity is not adjusted to ensure the same an objective lens transmittance for ultraviolet dye illumination. This is an important preference for a fluorescence system that uses CCD cameras.

## Declarations

### Author contribution statement

Vyacheslav M. Shkryl: Conceived and designed the experiments; Performed the experiments; Analyzed and interpreted the data; Contributed reagents, materials, analysis tools or data; Wrote the paper.

### Funding statement

This work was supported by the 10.13039/501100004742National Academy of Sciences of Ukraine.

### Competing interest statement

The authors declare no conflict of interest.

### Additional information

No additional information is available for this paper.

## References

[bib1] Barreto-Chang O.L., Dolmetsch R.E. (2009). Calcium imaging of cortical neurons using Fura-2 AM. J. Vis. Exp..

[bib2] Becker P.L., Fay F.S. (1987). Photobleaching of fura-2 and its effect on determination of calcium concentrations. Am. J. Physiol..

[bib4] Berridge M.J., Lipp P., Bootman M.D. (2000). The versatility and universality of calcium signalling. Nat. Rev. Mol. Cell Biol..

[bib5] Bootman M.D., Rietdorf K., Collins T., Walker S., Sanderson M. (2013). Ca^2+^-sensitive fluorescent dyes and intracellular Ca^2+^ imaging. Cold Spring Harb. Protoc..

[bib6] Bootman M.D., Rietdorf K., Collins T., Walker S., Sanderson M. (2013). Converting fluorescence data into Ca^2+^ concentration. Cold Spring Harb. Protoc..

[bib25] European convention, Strasburg (1986). European Convention for the Protection of Vertebrate Animals used for Experimental and other Scientific Purposes. https://www.coe.int/en/web/conventions/full-list/-/conventions/treaty/123.

[bib7] Grynkiewicz G., Poenie M., Tsien R.Y. (1985). A new generation of Ca^2+^ indicators with greatly improved fluorescence properties. J. Biol. Chem..

[bib8] Harbig K., Chance B., Kovach A.G., Reivich M. (1976). In vivo measurement of pyridine nucleotide fluorescence from cat brain cortex. J. Appl. Physiol..

[bib9] Heintzmann R., Pawley J.B. (2006). Structured illumination methods. Handbook of Biological Confocal Microscopy.

[bib10] Jerome W.G., Price R.L., Jerome W.G. (2011). Digital image capture for confocal microscopy. Basic Confocal Microscopy.

[bib12] Minta A., Kao J.P., Tsien R.Y. (1989). Fluorescent indicators for cytosolic calcium based on rhodamine and fluorescein chromophores. J. Biol. Chem..

[bib13] Neher E. (2013). Quantitative aspects of calcium fluorimetry. Cold Spring Harb. Protoc..

[bib14] Neher E. (1995). The use of fura-2 for estimating Ca buffers and Ca fluxes. Neuropharmacology.

[bib15] Paddle B.M. (1985). A cytoplasmic component of pyridine nucleotide fluorescence in rat diaphragm: evidence from comparisons with flavoprotein fluorescence. Pflueg. Arch. Eur. J. Physiol..

[bib16] Pozzan T., Rizzuto R., Volpe P., Meldolesi J. (1994). Molecular and cellular physiology of intracellular calcium stores. Physiol. Rev..

[bib17] Rios E., Stern M.D., Gonzalez A., Pizarro G., Shirokova N. (1999). Calcium release flux underlying Ca^2+^ sparks of frog skeletal muscle. J. Gen. Physiol..

[bib18] Scheenen W.J., Makings L.R., Gross L.R., Pozzan T., Tsien R.Y. (1996). Photodegradation of indo-1 and its effect on apparent Ca^2+^ concentrations. Chem. Biol..

[bib19] Shkryl V.M. (2017). Intracellular calcium fluxes in excitable cells. Neurophysiology.

[bib20] Shkryl V.M., Nikolaenko L.M., Kostyuk P.G., Lukyanetz E.A. (1999). High-threshold calcium channel activity in rat hippocampal neurones during hypoxia. Brain Res..

[bib23] Thomas D., Tovey S.C., Collins T.J., Bootman M.D., Berridge M.J., Lipp P. (2000). A comparison of fluorescent Ca^2+^ indicator properties and their use in measuring elementary and global Ca^2+^signals. Cell Calcium.

[bib21] Tsien R.Y., Pozzan T., Rink T.J. (1982). Calcium homeostasis in intact lymphocytes: cytoplasmic free calcium monitored with a new, intracellularly trapped fluorescent indicator. J. Cell Biol..

[bib22] Wokosin D.L., Loughrey C.M., Smith G.L. (2004). Characterization of a range of fura dyes with two-photon excitation. Biophys. J..

