# Supplementary material for: Error correction due to background subtraction in ratiometric calcium measurements with CCD camera
Source: Heliyon. 2020 Jun 24;6(6):e04180. doi: 10.1016/j.heliyon.2020.e04180 (PMC7322130; doi:10.1016/j.heliyon.2020.e04180)
Supplement: Supplementary_material [file mmc1.docx]

**Appendix A**

At rest, most neurons have an intracellular concentration of free Ca^2+^ about 50 – 100 nM ([Berridge, 1998](#_ENREF_3)). For well-known values of basal level of free Ca^2+^: $\left[ \mathrm{Ca}^{2+} \right]=Са_{0}$ and condition then the coefficient of the lost excitation power *C_0_* = 1, then:

$\frac{\left( F_{340}(0)-F_{340}^{min} \right)}{\left( F_{340}^{max}-F_{340}(0) \right)}K_{d}=Ca\_0$ (A1)

At rest level (Са_0 = 75 nM ([Korol et al., 2008](#_ENREF_11))): $F_{340}^{min}=F_{340}\left( 0 \right)-\frac{Ca\_0}{K_{d}}\cdot\left( F_{340}^{max}-F_{340}\left( 0 \right) \right)$ (A2)

$F_{340}^{max}$ can be estimated empirically as 20% of the peak Ca^2+^ transient: $F_{340}^{max}=$ $1.2 \cdot F_{340}(1)$ , where F_340_(1) is the value of Ca^2+^ transient at peak; F_340_(0) – rest level fluorescence.

Using this value we can write: $F_{340}^{min}=F_{340}(0)-\frac{75}{K_{d}}\cdot\left( 1.2 \cdot F_{340}(1) -F_{340}(0) \right)$ (A3)

To find the minimal and maximal fluorescence values of 380 nm light we will use the rest level value as F(0) and the Ca^2+^ transient value at the peak as F(1) for both wavelength. The following equations determine the minimum and maximum values for fluorescence at 380 nm light:

$F_{380}^{min}=\frac{F_{380}(1)\cdot\left( \frac{F_{340}\left( 1 \right)-F_{340}^{min}}{F_{340}^{max}-F_{340}\left( 1 \right)}+1 \right) {-F}_{380}(0)\cdot\left( \frac{F_{340}(0)-F_{340}^{min}}{F_{340}^{max}-F_{340}(0)}+1 \right)}{\frac{F_{340}(1)-F_{340}^{min}}{F_{340}^{max}-F_{340}(1)}-\frac{F_{340}(0)-F_{340}^{min}}{F_{340}^{max}-F_{340}(0)}}$ and

$F_{380}^{max}=F_{380}(0)\cdot\left( \frac{F_{340}(0)-F_{340}^{min}}{F_{340}^{max}-F_{340}(0)}+1 \right)-F_{380}^{min}\cdot\frac{F_{340}(0)-F_{340}^{min}}{F_{340}^{max}-F_{340}(0)}$ (A4)

References

Berridge M.J., Neuronal calcium signaling, Neuron 21 (1998) 13–26.

Korol T.Y., Korol S.V., Kostyuk E.P., Kostyuk P.G., β-amyloid-induced changes in calcium homeostasis in cultured hippocampal neurons of the rat, Neurophysiology 40 (2008) 6–9.
